# Cast Iron Parts Obtained in Ceramic Molds Produced by Binder Jetting 3D Printing—Morphological and Mechanical Characterization

**DOI:** 10.3390/ma14164502

**Published:** 2021-08-11

**Authors:** Răzvan Păcurar, Petru Berce, Ovidiu Nemeş, Diana-Irinel Băilă, Dan Sergiu Stan, Alexandru Oarcea, Florin Popişter, Cristina Miron Borzan, Sven Maricic, Stanislaw Legutko, Ancuţa Păcurar

**Affiliations:** 1 Department of Manufacturing Engineering, Faculty of Industrial Engineering, Robotics and Production Management, Technical University of Cluj-Napoca, B-dul Muncii 103-105, 400641 Cluj-Napoca, Romania; petru.berce@tcm.utcluj.ro (P.B.); cristina.borzan@tcm.utcluj.ro (C.M.B.); ancuta.costea@tcm.utcluj.ro (A.P.); 2 Department of Environmental Engineering and Sustainable Development Entrepreneurship, Faculty of Materials and Environmental Engineering, Technical University of Cluj-Napoca, B-dul Muncii 103-105, 400641 Cluj-Napoca, Romania; 3 Department of Manufacturing Engineering, Faculty of Industrial Engineering and Robotics, Polytechnic University of Bucharest, Splaiul Independenţei nr. 313, Sector 6, 060042 Bucharest, Romania; 4 Faculty of Automotive, Mechatronics and Mechanical Engineering, Technical University of Cluj-Napoca, B-dul Muncii 103-105, 400641 Cluj-Napoca, Romania; Sergiu.Stan@mdm.utcluj.ro (D.S.S.); Alexandru.Oarcea@mdm.utcluj.ro (A.O.); 5 Department of Design Engineering and Robotics, Faculty of Industrial Engineering, Robotics and Production Management, Technical University of Cluj-Napoca, B-dul Muncii 103-105, 400641 Cluj-Napoca, Romania; florin.popister@muri.utcluj.ro; 6 Institute for Science and Technology VISIO, Juraj Dobrila University of Pula, 52100 Pula, Croatia; smaricic@unipu.hr; 7 Faculty of Mechanical Engineering, Poznan University of Technology, 60-965 Poznan, Poland; stanislaw.legutko@put.poznan.pl

**Keywords:** binder jetting, 3D printing, iron casting, gravitational casting, finite element analysis

## Abstract

Mechanical behavior and characteristics of two different types of materials: cast iron with lamellar graphite EN-GJL-250 and cast iron with spheroidal graphite EN-GJS-400-15 which were cast in ceramic molds using gravitational casting method has considered in this research. The ceramic molds were obtained by 3D printing method. First, a finite element analysis was developed to determine Tresca and von Mises stresses and the deformations of the ceramic molds under an applied pressure of 25 MPa. Samples were produced by gravitational casting using two types of cast iron materials. Mechanical tests were made using samples produced from these two types of materials and microstructure analysis evaluation of fractured zones was realized by scanning electron microscopy. Obtained results were finally used for designing, developing, and producing of one ‘hydraulic block’ of a railway installation by the Benninger Guss company of Switzerland.

## 1. Introduction

It is well known that the mechanical properties of gray irons based on lamellar graphite are weaker than the ones of vermicular or spheroidal irons [1,2]. Beside these aspects it was determined that with the increase of graphite in the structure of the material, the thermal conductivity is significantly increased, especially in the case of gray irons with lamellar structure [3,4]. In this context, this material is preferred to be chosen for developing of different types of products that are subjected to thermal and mechanical stresses, even if the overall level of characteristics is lower as compared to the vermicular irons or gray spheroidal irons. Graphite is essential for the erosion behavior of iron cavities and understanding its formation mechanisms in realistic operating environments it is necessary to find solutions to improve the mechanical properties of the material in close correlation with the type of application for which the material is used [5,6,7]. Furthermore, the austenitizing temperature has a significant influence on the microstructure and mechanical characteristics of the material [8,9]. Analyzing the fracture zones of the samples by SEM (scanning electron microscopy) provides important information to determine how mechanical characteristics, such as tensile, flexural, or compression are mainly influenced by the type of casting processes of samples [10,11,12,13].

Cast iron melt is a complex poly-component solution. In terms of chemical composition of cast iron, solutions are characterized by high carbon content (up to 3.8%) and the content of silicon (up to 2.8%), manganese, sulfur, and phosphorus. The high content of carbon and silicon-iron melt differs significantly from the molten steel in terms of physical properties (such as viscosity, surface tension, and volume changes). Viscosity affects the property of flowability and, therefore, the iron mold filling by melt iron. The viscosity of the molten cast iron may complicate the casting and slag flowing out (because of vaccination melt). After [14], the reasonable values for these elements in the composition of melt iron EN GJL 250 used in automotive industry are 3.3%C, 1.8%Si, 0.7%Mn, 0.02%P, and 0.07%S. The starting material to produce the cast iron is pig iron.

Viscosity decreases with the increasing of melting temperature. Experiments showed that carbon and silicon significantly lower the viscosity and, thereby, improve the flowability of iron. Dynamic viscosity of lamellar graphite cast iron at 1310 °C is 2.65 × 10^−3^ Pa·s^−1^; the kinematic viscosity is 0.30 × 10^−6^ m^2^·s^−1^. With additions of alloying elements (Cu, Ni, W, V, and Mn) of over 1% content, the viscosity usually decreases. Surface tension of lamellar graphite cast iron at 1300 °C is 1.1 N·m^−1^. Volume changes are reflecting by changes of the specific volume. Flake graphite cast iron containing 3.5%C and 2.5%Si has a specific volume of 0.16 cm^3^·kg^−1^; it is an increase in the specific volume of 9.3%. Molten cast iron structure described according to the theory of chemical inhomogeneity due to added impurities.

At temperatures up to 1350 °C, there are practically no changes in the chemical composition of the melt iron. At higher temperatures, the carbon and manganese are burned. At temperatures of 1450 °C, the value of the burn carbon is to 0.20% per hour; by silicon and manganese, the change is slight. At temperatures higher than 1450 °C, the silicon and manganese changes arise. During casting, it is important that the ladle was warmed to approximately 600 °C [14].

Even if there are several technological methods of casting, sand casting and investment casting methods remain some of the most used technological methods of casting due to the fact that these methods are not very expensive, especially in the case when different types of mixing alloys are required to be rapidly tested or produced [15,16]. The mold made of sand material allows rapid cooling and solidifying of the liquid material that is being cast, the realized product being easily extracted from the mold by braking the ceramic form, post-processing operations being possible to be applied on the realized product in order to improve the surface roughness as well [17,18]. In case of mixing alloys, there were significant research that were made for studying the influence of different types of materials in the matrix of the iron-casting alloys on the mechanical behavior of realized samples [19,20]. The possibility of producing compacted graphite iron it was noticed that has a significant influence in obtaining higher mechanical strength as compared to the case when gray cast iron is used and this offers great advantage in the case when mechanical parts for automotive or railway sector are necessary to be developed and produced [21,22,23]. Also, in the case of ceramic molds that are used for producing the iron parts by casting, there were significant studies performed in this domain. The potential of using recycled sand materials mixed with bentonite and water in different ratios had an influence on the mechanical and thermal resistance of the mold being produced [24,25]. The manufacturing methods of the ceramic mold in concordance to the characteristics of the mixing materials have one significant influence on the microstructural defects (such as cracks, pores, etc.), as other researchers observed by analyzing the parts and the molds being produced on the scanning electron microscopy analyses [26,27].

The appearance of 3D printing and rapid tooling in the last decade, correlated with the requests from the industrial sector for rapid development of products and producing of different parts or testing new materials, represented a significant step that was made forward in this direction. There are several methods that are currently used, and the most known are the fused deposition modeling (FDM), selective laser sintering (SLS), stereolithography (SLA), etc. [28,29,30,31,32,33]. Binder jet 3D printing, also known as “powder bed and inkjet” and “drop-on-powder” printing is one of the most used method that can be used for realizing of ceramic parts, but also ceramic molds that can be further on used for casting processes of metallic parts (e.g., gravitational casting, investment casting, etc.). [34,35]. Parts printed using the binder jetting process are porous and have an unfinished surface, unlike selective laser sintering, where the powders are not physically melted and are joined by a binding agent [36]. While the usage of a binding agent allows high melting temperature, these parts require additional post-processing and more time than it takes to print the part, such as curing, sintering, and additional finishing [37,38]. Even if the binder-jetted parts are weaker than those printed by selective laser sintering, they are an adequate solution and are recommended to be used for developing and producing low-cost metallic parts, such as iron materials [39].

The research presented in this paper is focused on the product ‘hydraulic block’ that was designed, developed, tested, and finally produced with the Benninger Guss AG company of Uzwil, Switzerland equipment [40]. The company is using gravitational method DGP (Digital Production of Casting) for realizing prototypes and complex products in small batches made of different types of materials (gray spheroidal and lamellar irons, etc). Gravitational casting is among the oldest known processes for fabricating metals and metal alloys. The melted metal is poured from a crucible into a mold under only the force of gravity, without the use of pressurized gases, vacuums, or centrifugal force. Molds made of ceramic or sand material are used since these materials are generally easy to shape (unlike\steel), do not break down when suddenly are exposed to high temperatures, do not deform easily and are widely available. The company is testing Binder Jetting 3D printing technology for realizing ceramic molds for obtaining test samples and products developing for automotive and railroad industrial sectors.

## 2. Materials and Methods

The main goal of the research is to design and manufacture a ‘hydraulic block’ for a railway installation, which is developed within the Benninger Guss company from Switzerland. This part represents a complex spatial shape and was modeled using the CAD software Dassault Systems SolidWorks 2015 (Vélizy-Villacoublay, France). The parts were realized by casting from two types of cast iron materials: cast iron with lamellar graphite EN-GJL-250 and cast iron with spheroidal graphite EN-GJS-400-15. The product hydraulic block of the railway installation is manufactured in cast iron, because needs to present good mechanical resistance, good hardness, and dampen vibrations.

**Figure 1:**
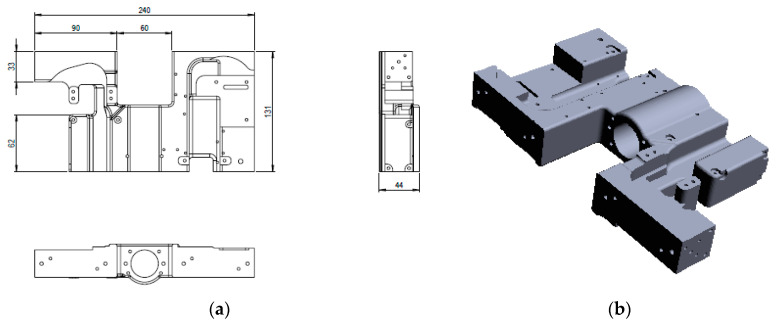
Hydraulic block of one railway installation: (**a**) 2D technical drawing; (**b**) 3D CAD model.

**Figure 2:**
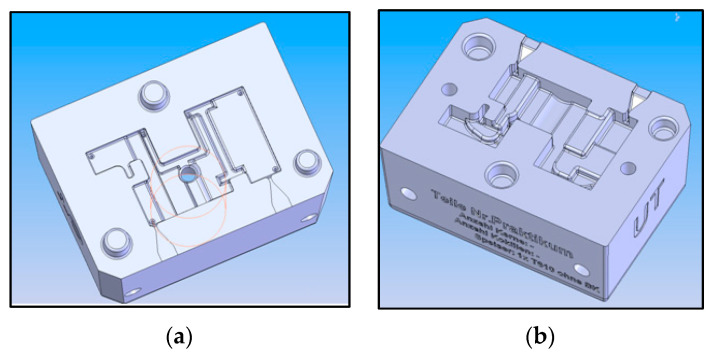
Ceramic molds for the hydraulic block of the railway installation: (**a**) Upper mold/die; (**b**) Lower mold/die.

The ‘hydraulic block’ CAD model, which is presented in Figure 1 was further on subjected to a finite element analysis (FEA) using ABAQUS 6.14 (Vélizy-Villacoublay, France), in order to determine the distribution of stresses and deformations within the hydraulic block as part of the railway installation exposed to static stresses. The parts made by casting were raw parts, and then mechanically processed to obtain the finished components. Gravitational casting of cast iron parts was done in ceramic molds, which were previously made on a rapid prototyping machine (S-Max ExOne—North Huntingdon, PA, USA) using DGP (Digital Guss Production) technology. The mold cavity was made at a size of 1% larger than the size of the part that resulted from the casting because the material is shrinking. Shrinkage is a natural property that can cause twists, internal gaps, internal stresses, or cracks on the limits. For parts manufactured by DGP, the following materials are used: quartz sand SiO_2_—Sand Casting 3D Printing, activator (chemical solution), inorganic binder, cleaning materials. The sand used to make the parts is stored in two bunkers. The sand is mixed with the activator (a hardener) in the machine mixer which has a capacity of 8 kg. The molds needed for the DGP technology were designed in SolidWorks CAD software as shown in Figure 2.

The inorganic binder is a water-based, alkali-silicate binder requiring additional post-curing at elevated temperatures. Additional proprietary powder and fluid additives are incorporated into the process to optimize printed core and mold strengths as well as high-temperature casting properties.

The molds obtained by binder-jetting technology, using sand powder and binder, are temporary and are used only to obtain one product, but the quality and detail of the surfaces of cast parts are superior to those obtained by classical variants of casting in temporary forms, and the roughness of the semi-finished product obtained by casting is very low (Ra = 3.2 μm). Basically, the cast part does not require other further processing to obtain this roughness. This casting technology, using temporary shapes made by binder-jetting technology, is recommended to be used for making prototypes and parts in the aeronautical industry, where a fine roughness of the cast surfaces are required.

**Figure 3:**
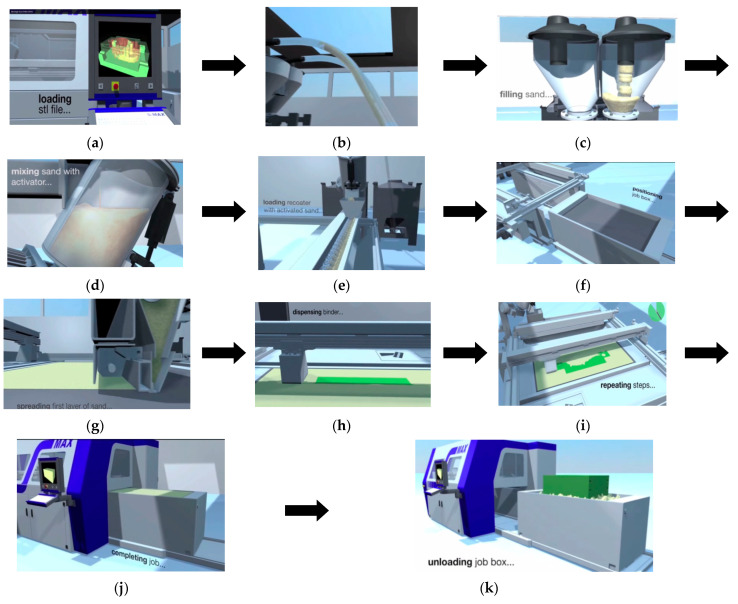
Stages of making ceramic molds by 3D printing: (**a**) Importing the “stl” model in the Rapix3D program; (**b**) Suppling sand from machine bunkers; (**c**) Loading with sand the small bunkers above the machine; (**d**) Mixing the sand with the activator in the machine mixer; (**e**) Transport of the mixture through the injecting screw; (**f**) Positioning the machine table in the workspace; (**g**) Laying the first layer of ceramic material; (**h**) Binder dispersion; (**i**) Repeating Steps 7 and 8; (**j**) Ending the manufacturing process of ceramic parts; (**k**) Removing parts from the workspace.

**Figure 4:**
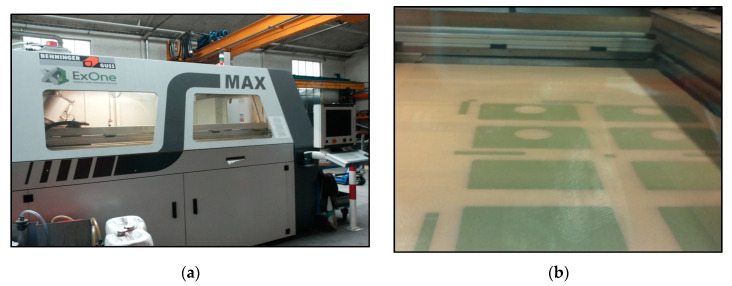
Manufacturing of ceramic molds for the construction of a hydraulic block used in railway installation: (**a**) S-Max ExOne machine for the manufacturing of ceramic parts; (**b**) Image from the manufacturing process of ceramic molds on the S-Max ExOne machine.

In the next stage, the CAD models of the molds were imported into the Rapix3D 2015 program (FORWISS Passau, Germany) with which the ExOne machine (North Huntingdon, PA, USA) works. Inside this program, an arrangement was made for the parts to manufacture, to fit in the machine workspace (job box). The space between the parts must always be at least 2–3 mm, otherwise there is a risk that they will stick together. Figure 3 shows the manufacturing steps of the ceramic molds:

The Rapix3D program calculates the number of layers of sand required to make the parts and to estimate the manufacturing time. The thickness of the sand layer was 0.3 mm and 750 layers are needed to reach the highest point in the workspace (225 mm). To manufacture the molds, the S-Max ExOne machine (Figure 4a) worked for 7 h and 17 min and used 4.8 kg of fresh sand mixed with 3.2 kg of used sand and 0.35% binder. The machine is equipped with two sensors that constantly measure the height of the sand. The equipment also has a thermometer that indicates the temperature inside the machine, which should be about 30 °C during the entire manufacturing operation. A suggestive image during the manufacturing of the ceramic molds on the S-Max ExOne machine is shown in Figure 4b.

**Figure 5:**
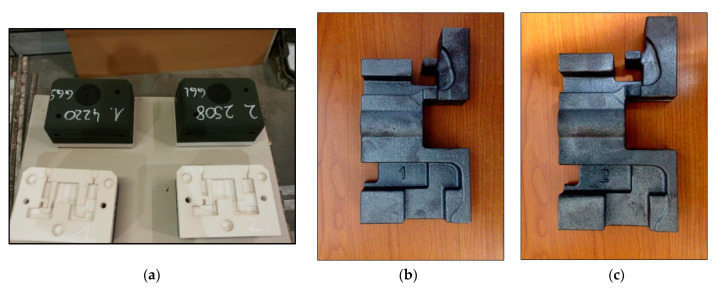
Molding plates and parts (**a**) ceramic molds used for casting the iron parts; (**b**) gray cast iron part with lamellar graphite type EN-GJL-250 resulting from casting and cleaning; (**c**) cast iron part with spheroidal graphite type EN-GJS-400-15 resulting from casting and cleaning.

After all the layers of ceramic material have been deposited and hardened, the machine table moves out of the workspace so that the parts can be removed and cleaned. For the active surfaces of the molds to have a finer and more uniform surface, the parts were inserted in a recipient with coating material, and then was left to dry for several hours. The ceramic molds used for casting cast iron parts are shown in Figure 5a.

In this research, for the manufacturing of the cast iron parts presented in Figure 5b, the casting in ceramic forms was used. The ceramic molds were made on the ExOne machine from the Benninger Guss AG company (Uzwil, Switzerland).

Benninger Guss ovens are 5-ton induction furnaces from which the melt material is poured into one-ton casting pots. To obtain spheroidal graphite cast irons it was necessary to introduce a quantity of MgSi and steel scraps into the casting pot. A lid was placed over the pouring pot and held for 1 min to produce the Mg reaction. Before casting, the melting temperature, which must be between 1360 °C and 1400 °C, was checked. The measured temperature was 1400 °C. After casting, the cast iron parts were allowed to cool in the air for 1 h and 30 min, after which the casting tree was removed and the parts were cleaned by blasting.

**Figure 6:**
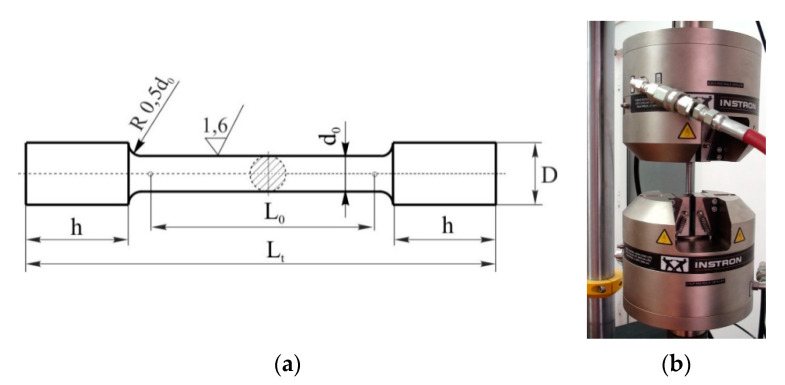
Tensile test experiment (**a**) standard test specimen of circular section where h = 10 mm, L_0_ = 60 mm, Lt = 140 mm, d_0_ = 4 mm, and D = 12 mm; (**b**) attaching the test samples to the jaws of the hydraulically actuated machine.

To determine the fields of deformations and stresses that appear inside the hydraulic block manufactured by the 3D printing method, tensile tests were previously performed using 20 samples produced by casting, using cast iron spheroidal graphite type EN-GJS-400-15 and lamellar cast iron type EN-GJL-250 (see Figure 6a). The conditions for performing the tensile test and the interpretation of the results were those specified in the standard SR EN 10002-1:1990. The test samples were performed on Instron 8801 test machine (Norwood, MA, USA), which has a loading capacity of up to 100 kN (see Figure 6b).

An analysis was also performed by using an electron microscope on the fracture area of the test samples subjected to the tensile test. The analysis of the metallographic samples was performed with the help of the Zeiss Jenoptik AXIO microscope (Oberkochen, Germany). The preparation of the metallographic samples was done according to the indications of STAS 4203-74 and involved of the following operations: preparing of the samples by: sanding, polishing, and the metallographic attack. Sanding of the samples was performed with the help of meta-paper papers (abrasive carbide particles are silicon on paper or cloth). The samples were sanded manually, using eight metallographic papers starting from granulation 150 to granulation 800. At each change of paper, the sample was cleaned and rotated by 90°, so that the new residues form a right angle with the previous ones. After grinding, the samples were washed under running water to remove the residues of the abrasive. The polishing of the samples was performed on the polishing machine equipped with a rotating disc on which a thick cloth felt was fixed. The polishing agent used was Al_2_O_3_ alumina. The polished samples were washed with water and degreased with alcohol at the end.

The chemical analysis of the samples made from the two types of cast iron was done with a spectrometer called SPECTROMAXx (Kleve, Germany). For each sample it was calculated the carbon equivalent EC (or degree of carbon saturation) indicating the nature of the cast iron. The SPECTROMAXx Stationary Metal Analyzer is mainly used for testing material in foundries and for the entry and exit inspections of the metal industry around the globe. The spectrometer determines all the elements used in the metal industry, including the analysis of traces of carbon, phosphorus, sulfur, nitrogen. The spectrometer independently monitors all operating parameters. It dynamically determines the time required for the measurements based on the properties of the sample to be analyzed and shows when it is necessary to clean the spark stand depending on the type of sample to be examined.

**Figure 7:**
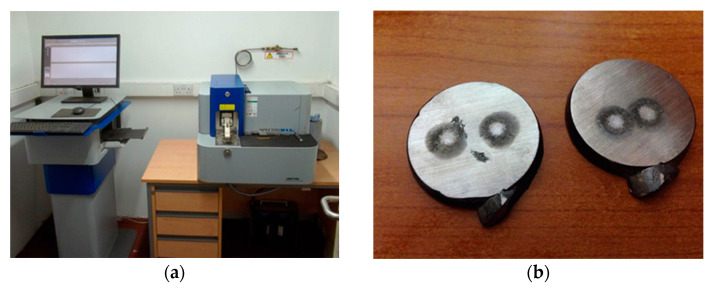
Equipment and samples used for chemical analysis (**a**) the SPECTROMAXx chemical analysis spectrometer of Benninger Guss AG. (**b**) Surface of the samples after chemical analysis performed with the spectrometer.

For the chemical analysis, several areas of the surfaces were considered by the spectrometer in the case of each samples to be analyzed (Figure 7a). For a more precise analysis, two points of measurement were considered on the surface of the samples (Figure 7b).

## 3. Results

### 3.1. Tensile Test of Cast Iron Samples Made by Gravitational Casting in Ceramic Molds Made of 3D Printing

The results of the tests carried out on the samples of gray cast iron with lamellar graphite type EN-GJL-250 are presented in Table 1.

The specimen to whom the greatest force was applied was the specimen number 1, and its breaking process resulted in the following characteristics: Maximum force = 22,103.12 (N); Specific deformation = 0.01245 (mm); Tensile strength = 275.34 (MPa); Elongation at break = 1.9 (%); Elasticity Modulus = 37,039.12 (MPa).

The sample to which the least force was applied was the sample number 4, and its breaking process resulted in the following characteristics: Maximum force = 21,074.23 (N); Specific deformation = 0.01089 (mm); Tensile strength = 273.75 (MPa); Elongation at break = 0.9 (%); Elasticity Modulus = 37,002.67 (MPa).

**Figure 8:**
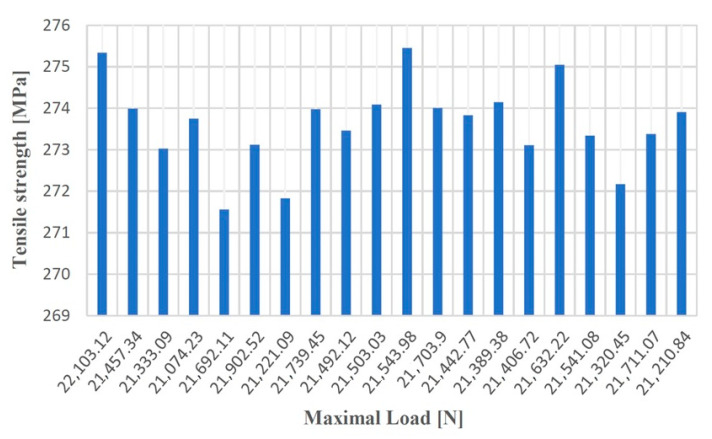
Tensile strength values according to the maximum forces applied to the 20 test samples made of cast iron with lamellar graphite type EN-GJL-250.

**Figure 9:**
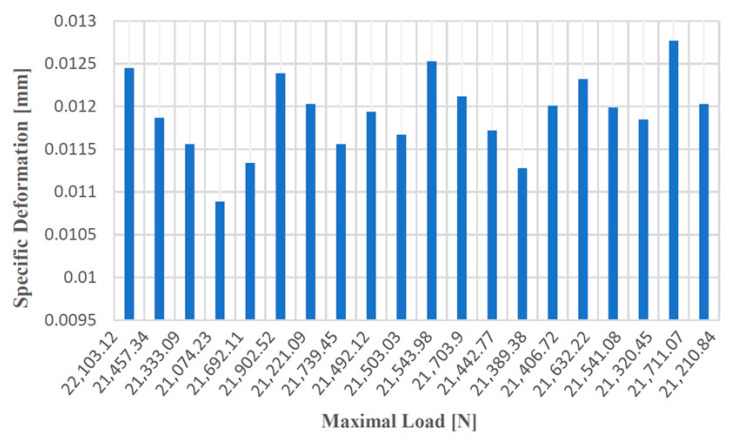
Values of the specific deformation according to the maximum forces applied to the 20 samples made of cast iron with lamellar graphite type EN-GJL-250.

**Figure 10:**
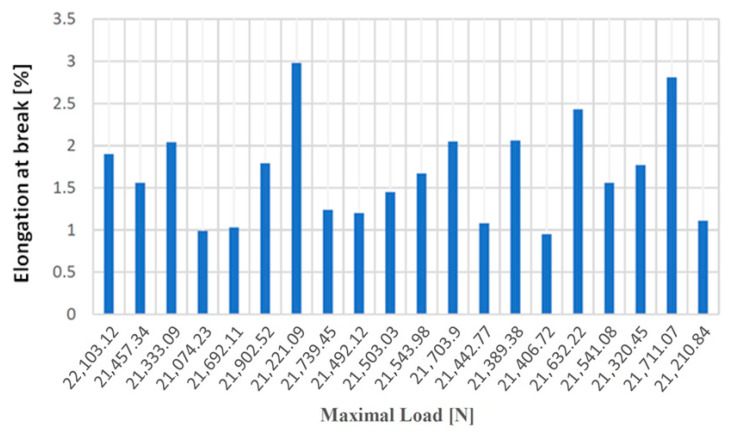
Elongation at break values at failure depending on the maximum forces applied to the 20 samples made of cast iron with lamellar graphite type EN-GJL-250.

**Figure 11:**
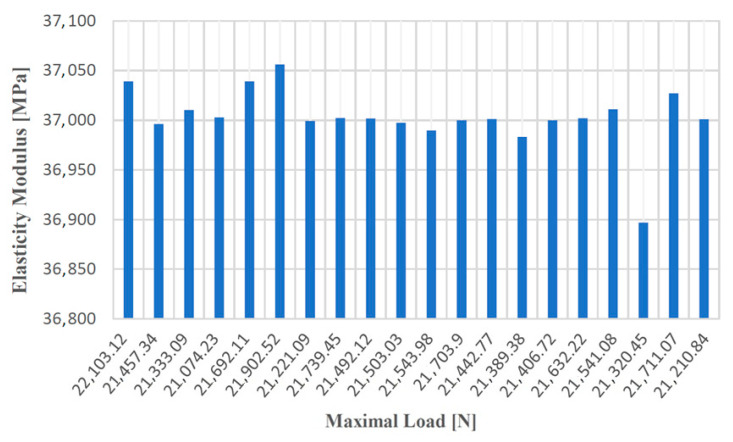
Values of the modulus of elasticity according to the maximum forces applied to the 20 samples made of cast iron with lamellar graphite type EN-GJL-250.

Figure 8, Figure 9, Figure 10 and Figure 11 show the diagrams of variation of the tensile strength, specific deformation, elongation at break and modulus of elasticity depending on the values of maximum forces applied to the 20 samples of gray cast iron with lamellar graphite type EN-GJL-250.

In the case of the tensile test for test samples made from cast iron with spheroidal graphite type EN-GJS-400-15, the resulting values are given in Table 2.

The sample to which the greatest force was applied was the sample number 13, and from its breaking process resulted the following characteristics: Maximum force = 35,721.03 (N); Specific deformation = 0.16533 (mm); Tensile strength = 417.57321 (MPa); Elongation at break = 22.03 (%); Elasticity Modulus = 54,288.51 (MPa).

The sample to which the least force was applied was the sample number 14, and its breaking process resulted in the following characteristics: Maximum force = 35,121.17 (N); Specific deformation = 0.16244 (mm); Tensile strength = 417.56922 (MPa); Elongation at break = 22.41 (%); Elasticity Modulus = 54,244.06 (MPa).

**Figure 12:**
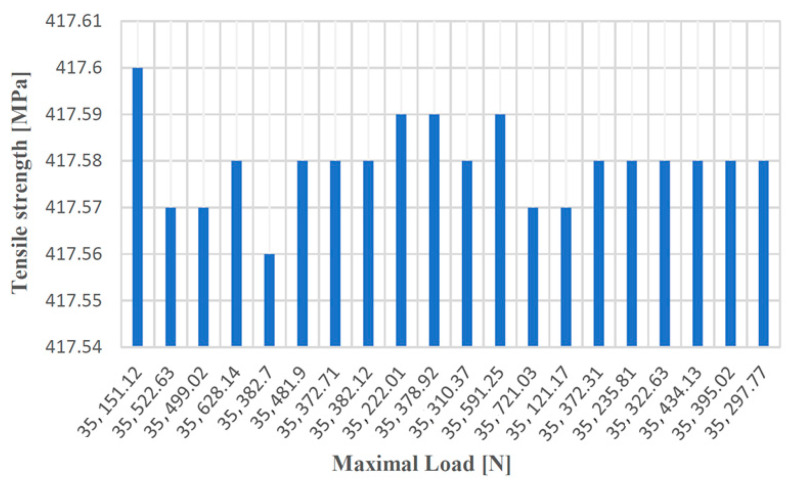
Values of the tensile strength according to the maximum forces applied to the 20 samples made of cast iron with spheroidal graphite type EN-GJS-400-15.

**Figure 13:**
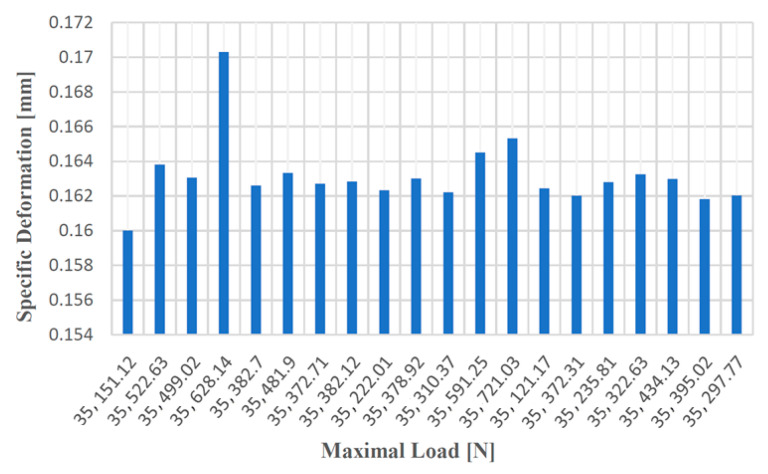
Values of the specific deformation according to the maximum forces applied to the 20 samples made of cast iron with spheroidal graphite of type EN-GJS-400-15.

**Figure 14:**
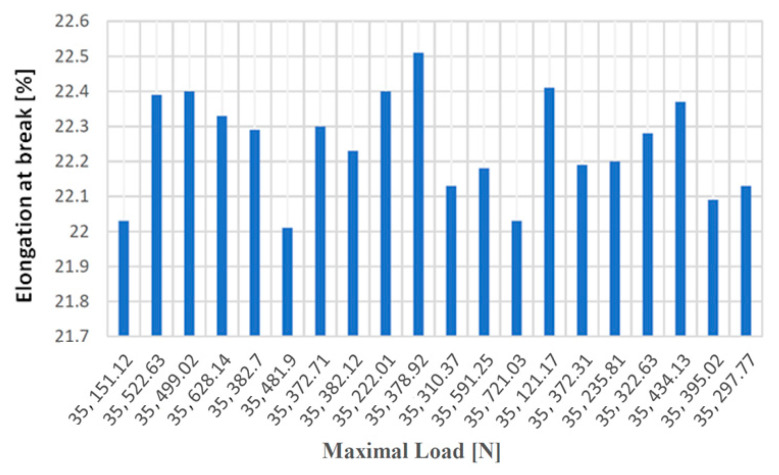
Elongation at break values at failure depending on the maximum forces applied to the 20 samples made of cast iron with lamellar graphite type EN-GJS-400-15.

**Figure 15:**
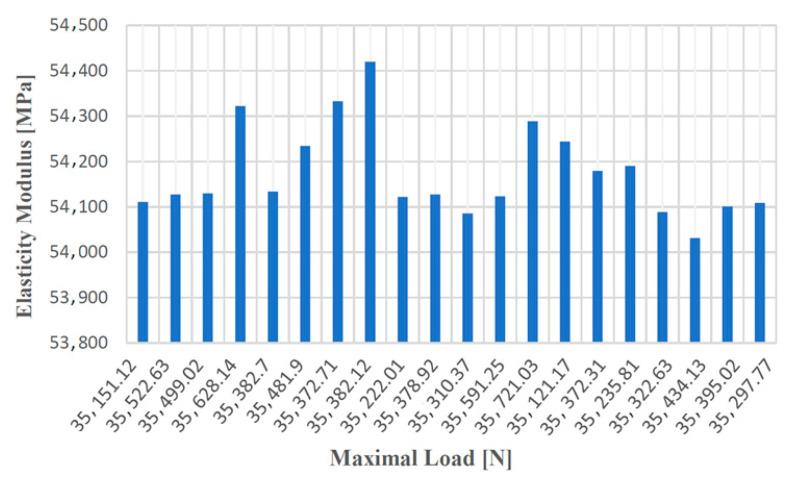
Values of the modulus of elasticity according to the maximum forces applied to the 20 samples made of cast iron with spheroidal graphite type EN-GJS-400-15.

Figure 12, Figure 13, Figure 14 and Figure 15 show the diagrams of variation of tensile strength, specific deformation, elongation at break and modulus of elasticity depending on the values of maximum forces applied to the 20 cast iron testing samples with spheroidal graphite type EN-GJS-400-15.

The values of the elongation at failure depending on the maximum forces applied to the 20 samples made of cast iron with spheroidal graphite type EN-GJS-400-15.

An average tensile strength value, in the case of the test of samples made of gray cast iron with lamellar graphite type EN-GJL-250, was considered for sample number 9, for which the following values were obtained: Maximum force = 21,492.12 (N); Specific deformation = 0.016272 (mm); Tensile strength = 273.46 (MPa); Elongation at break = 1.2 (%); Elasticity Modulus = 37,001.83 (MPa).

In the case of testing samples made of cast iron with spheroidal graphite type EN-GJS-400-15, the average value between the applied forces was considered for specimen number 7, for which the following values were obtained: Maximum force = 35,374.71 (N); Specific deformation = 0.01194 (mm); Tensile strength = 417.58017 (MPa); Elongation at break = 22.3 (%); Elasticity Modulus = 54,333.14 (MPa).

**Figure 16:**
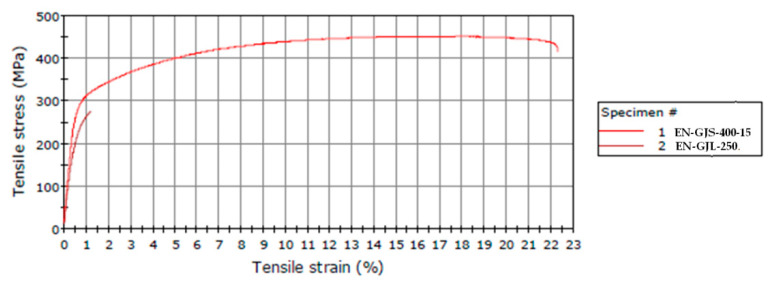
Variation of the tensile strength as a function of elongation at failure in the case of the two testing samples representing the average values of the tests.

The diagram in Figure 16 shows the variation of the tensile strength depending on the elongation at failure, for the two samples representing the average values of the tests, in the case of the two types of materials (EN-GJL-250, EN-GJS-400-15).

In the diagram shown in Figure 16, curve 1 is the characteristic curve for the sample made of cast iron with spheroidal graphite type EN-GJS-400-15, and curve 2 is the characteristic curve for the sample made of cast iron with lamellar graphite type EN-GJL-250. Figure 16 shows that the cast iron sample with lamellar graphite type EN-GJL-250 suffered a sudden and rapid fracture near one of the ends, the fracture being a characteristic of cast irons, which are stiff but fragile material. Fragile fracture produces a normal separation section on the axis. In contrast, the sample made of spheroidal graphite cast iron underwent an elongation of 22.3% before breaking, a phenomenon that is not characteristic of cast irons. This elongation may be due to the speed of stress, because the slower the loading is, the higher the elongation at break would be.

### 3.2. Finite Element Analysis Performed in the Abaqus Program of the “Hydraulic Block” Part, Taking into Consideration the Results Obtained Concerning the Tensile Test of the Cast Iron Test Samples

The purpose of this analysis by using the finite element method (FEA) was to determine the fields of deformations and stresses that occur inside the hydraulic block in a railway installation, subjected to static stresses. This component has a complex spatial geometry and has been considered to be made of two types of cast iron: EN-GJL-250 (gray cast iron with lamellar graphite) and EN-GJS-400-15 (gray cast iron with spheroidal graphite). The importing of the geometric model of the part (made in SolidWorks) was made in the form of a ParaSolid file with the extension “x_t”. Further on, in order to define the material characteristics, a new type of material was defined and considered for a body with elastic behavior to which the values of Young’s Mode and Poisson’s ratio were assigned for these two tpes of iron materials. The characteristics of the two analyzed materials were those from the SolidWorks program library, as well as those determined following the mechanical tensile test of the cast iron test samples that are presented in Table 3.

**Figure 17:**
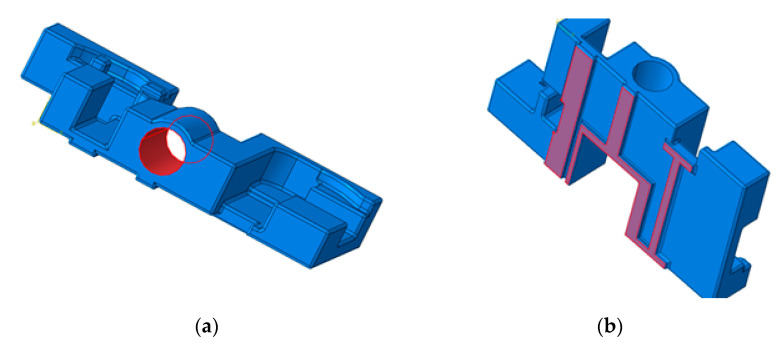
Constraints applied to the part model: (**a**) Application of a pressure of 25 MPa (250 bar) on the inner surface of the hydraulic cylinder; (**b**) Application of the kinematic constraints of embedding type which materialize the fixing of the hydraulic block on the support.

In order to establish the technological constraints, a pressure of 250 bar was considered (normal value used in hydraulic applications (value that the hydraulic agent exerts on the part) has been considered as shown in Figure 17a). The application of the pressure of 25 MPa (250 bar) was performed on the inner surface of the hydraulic cylinder. It was then defined the built-in kinematic constraints that materialize the fixing of the hydraulic block made on the support (Figure 17b).

Generating of the finite element mesh was further on performed. 91,261 tetrahedral elements with C3D10M second degree interpolation polynomial and a total number of 141,607 nodes were generated.

**Figure 18:**
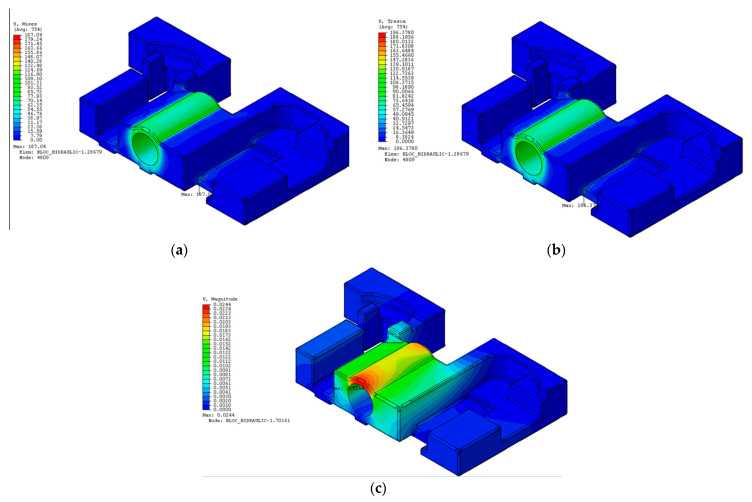
Results of the finite element analysis: (**a**) von Mises equivalent stress distribution; (**b**) Tresca equivalent stress distribution; (**c**) deformation distribution.

Regarding the von Mises equivalent stress distribution, the maximum value shown on the diagram in Figure 18a was

(1) $$\sigma_{ech}max=187.04\;\mathrm{MPa},$$

This stress level corresponds to the following value of the safety factor C with respect to the tensile strength *R_m_* = 250 MPa (case of cast iron EN-GJL-250)

(2) $$\frac{R_{m}}{\sigma_{ech}max}=\frac{250}{187.04}=1.34,$$

In practice, many specialists prefer the equivalent Tresca stress characteristic instead of the von Mises characteristic, considering that the Tresca model is more realistic and describes much clear the behavior of brittle materials such as cast irons.

The maximum value highlighted on the diagram in Figure 18b is

(3) $$C=\frac{R_{m}}{\sigma_{ech}max}=\frac{250}{196.378}=1.27,$$

This level of stress corresponds to the following value of the safety factor *C* with respect to the tensile strength *R_m_* = 250 MPa is:

The most significant displacement was noticed at cylinder liners with a value of 0.0244 mm (Figure 18c). In general, these displacements are within the limits tolerated by the piston seals in the cylinder. This level of deformation can possibly be taken into account when analyzing the overall stiffness of the hydraulic block. In the case of EN-GJS-400-15 cast iron, which has the Young’s modulus and the Poisson’s ratio equal to the values of the EN-GJL-250 cast iron, the only difference is given by the higher mechanical strength, namely *R_m_* = 400 MPa, which will determine a different (higher) safety factor than the one resulting in the case of lamellar cast iron.

For the von Mises stress, at which $\sigma_{ech}max=187.04\;\mathrm{MPa}$, the safety factor is

(4) $$C=\frac{R_{m}}{\sigma_{ech}max}=\frac{400}{187.04}=2.14,$$

For the Tresca stress, at which $\sigma_{ech}max=196.37\;\mathrm{MPa}$, the safety factor is

(5) $$C=\frac{R_{m}}{\sigma_{ech}max}=\frac{400}{196.37}=2.03,$$

From Equations (4) and (5) it can be concluded that the designing of the hydraulic block made of cast iron EN-GJL-250 does not guarantee a safety factor of at least 1.5 (normal value taken into consideration in hydraulic applications) [41], this condition being ensured only for cast iron EN-GJS-400-15. In the case of the finite element analysis of the ‘hydraulic block’ part made of cast iron with lamellar graphite type EN-GJL-250, the stress value was lower ($\sigma_{ech}max$ = 196.378 MPa) than in the case of testing samples of the same type of cast iron on the Instron 8801 machine with a load capacity of 100 KN ($\sigma_{ech}max$ = 275.45 MPa). On the other hand, it was found that the cast iron samples made with spheroidal graphite type EN-GJS-400-15, but also those made of cast iron with lamellar graphite type EN-GJL-250, tested at tensile has suffered higher deformations (max 0.16533 mm) than the values resulting from the finite element analysis (max 0.0244 mm).

These differences between the two types of analyses are due to the characteristics of materials, which, in the case of finite element analysis, were taken from the SolidWorks material library and because of the elastic modulus and Poisson’s ratio, which were taken the same for the two types of cast iron. On the other hand, the tensile test performed on the samples made of two types of cast iron emphaszied that the elastic modulus was different than those which were considered for the SolidWorks material library, as can be seen from Table 1 and Table 2. This explains the identical results given by the finite element analysis of the two types of cast iron, because for their analysis only the modulus of elasticity and the Poisson’s ratio were required as input data (these being the same for both types of cast iron).

### 3.3. Microstructural and Chemical Analysis of Samples of Cast Iron with Lamellar Graphite Type EN-GJL-250 and Cast Iron with Spheroidal Graphite Type EN-GJS-400-15

**Figure 19:**
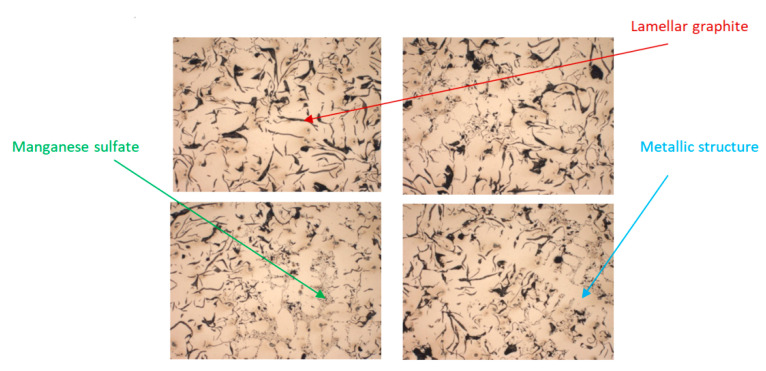
Microstructures of cast iron samples with lamellar graphite type EN-GJL-250, without metallographic attack at 100× magnification.

The analysis of the metallographic samples was performed with the help of the Zeiss Jenoptik AXIO microscope from the laboratory of Benninger Guss AG, used for the analysis of the cast iron samples made with lamellar graphite type EN-GJL-250 and the cast iron made with spheroidal graphite type EN-GJS-400-15 after the tensile test (breaking area). Figure 19 shows SEM images of cast iron samples of the samples made of lamellar graphite type EN-GJL-250, without metallographic attack.

**Figure 20:**
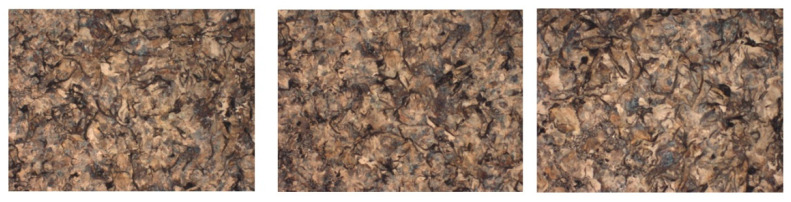
Microstructure of cast iron samples with lamellar graphite type EN-GJL-250, after metallographic attack at 100× magnification.

The presence of manganese in cast iron gives it higher level of hardness. The metallographic attack of the samples was performed to determine the amount of pearlite and ferrite in the cast iron structure with lamellar graphite type EN-GJL-250. Representative images captured under a microscope at 100× magnification are shown in Figure 20. The acid used for the metallographic attack of the samples was nitric acid (HNO_3_).

The images from Figure 20 showed that the structure of EN-GJL-250 lamellar graphite cast iron is 100% pearlite, in the form of lamellae. Pearlite gray cast irons have the best properties at static and dynamic stresses, a very good machinability by cutting and a high wear resistance.

**Figure 21:**
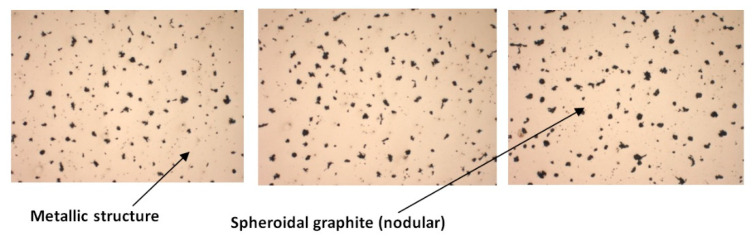
Microstructure of cast iron samples with spheroidal graphite type EN-GJS-400-15, without metallographic attack at 100× magnification.

Figure 21 shows SEM images (with one magnification of 100×) for the cast iron samples realized with spheroidal graphite type EN-GJS-400-15, without metallographic attack.

**Figure 22:**
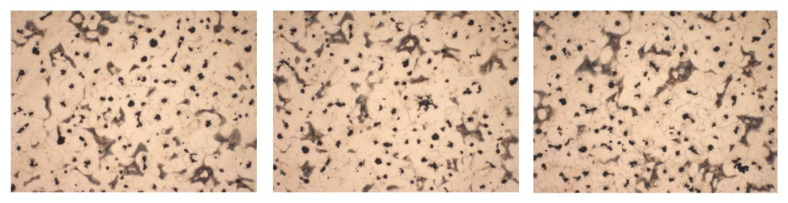
Microstructure of cast iron samples with spheroidal graphite type EN-GJS-400-15, after metallographic attack at 100× magnification.

Figure 22 shows SEM images after the metallographic attack with nitric acid (HNO_3_). From the analysis of these images, it was found that the structure of the studied sample has 20% pearlite and 80% ferrite. Ferrite-pearlite gray cast irons are the most common for foundry. The nodularization of graphite favors the improvement of both the mechanical strength and the plasticity of cast iron [42].

For the analysis of cast iron metallographic samples with spheroidal graphite type EN-GJS-400-15, a more detailed analysis was performed in the AMGUSS program 2015 version (Berlin, Germany).

**Figure 23:**
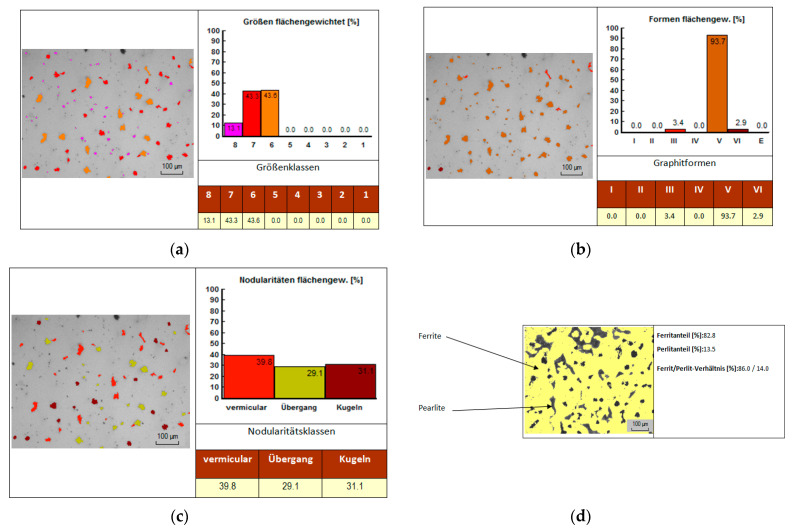
Graphite size analysis using AMGUSS analysis software: (**a**) Graphite size analysis using AMGUSS analysis software; (**b**) Graphite nodule shape analysis using AMGUSS analysis software; (**c**) Graphite nodularity analysis using AMGUSS analysis software; (**d**) Analysis of ferrite and pearlite concentration in the cast iron structure of type EN-GJS-400-15 using AMGUSS analysis software.

This analysis generated results regarding the size of graphite (Figure 23a), the shape of graphite (Figure 23b), nodularity (Figure 23c) and the concentration of ferrite and pearlite in the cast iron structure type EN-GJS-400-15 is shown in Figure 23d. The results generated by the analysis software are in accordance with DIN EN ISO 945-1.

From the graph of Figure 23a it was found that the size of graphite spheres fits in the standard size 6, because most of the graphite (43.6%) has this size. From the graph of Figure 23b it was highlighted that most of the graphite nodules (93.7%) were in the standard V shape, a shape that is very close to the circular shape. From the graph shown in Figure 23c it was found that graphite particles can be vermicular, spheroid or a combination of the two. The largest share (39.8%) has vermicular graphite nodules, and 31.1% of the nodules are spheroids. The vermicular shape of graphite allows a higher susceptibility to dissolution in the metal matrix. From Figure 23d it was observed that the percentages previously established for the metallographic analysis of the sample made of cast iron EN-GJS-400-15 after the chemical attack, namely 80% ferrite and 20% pearlite are very close to those given by the AMGUSS analysis (82.8% ferrite and 13.5% pearlite), which indicates a correct reading of the images under microscope.

The chemical analysis of the samples made from the two types of cast iron studied was done with a spectrometer called SPECTROMAXx. The chemical analysis showed the following values shown in Table 4 and Table 5. In the Table 4 and in the Table 5, are presented the chemical analysis of the sample made of gray cast iron type EN GJL 250 and EN-GJS-400-15, including the chemical percentages for P[%] and S[%]. The normal chemical composition cast iron EN GJL 250 should be C = 2.8–3.3%, Si = 1.2–1.7%, Mn = 0.8–1.2%, P ≤ 0.15%, S ≤ 0.12% [43]. In Table 4, can observed that, the 0.0127%P and 0.0113%S are in normal concentration. The reasonable value for cast iron EN-GJS-400-15 are (2.5–3.8)%C, (0.5–2.5)%Si, (0.2–0.5)%Mn, P ≤ 0.08% and S ≤ 0.02%. In Table 5, can remarked that, the 0.0127%P and 0.0113%S are in normal concentration [44].

Based on the values resulting from the chemical analyzes for the two types of cast iron, the EC carbon equivalent (or degree of carbon saturation) indicating the nature of the cast iron was calculated for each. The carbon equivalent is calculated from relation (6) [45]

(6) EC=C+0.31·S+0.27·P [%],

where *C* represents the amount of carbon, *S* represents the amount of sulfur and *P* represents the amount of phosphorus. If *EC* = 2.08 ÷ 4.26%, then cast iron is hypoeutectic. If *EC* = 4.26%, then cast iron is eutectic. If *EC* > 4.26%, then cast iron is hypereutectic. In the case of EN-GJL-250 cast iron, the value of the carbon equivalent was *EC* = 3.82%, so it is a hypoeutectic cast iron, and for the EN-GJS-400-15 type cast iron, the value of the carbon equivalent was *EC* = 4.27%, so a hypereutectic cast iron.

From the two analyses it was possible to observe the amount of magnesium required for the formation of cast iron with spheroidal graphite type EN-GJS-400-15, having a much higher value (0.0430%) than in the case of cast iron with lamellar graphite type EN-GJL-250 (0.0030). Nodular (spheroidal) graphite castings have better strength characteristics because coarse separations of lamellar graphite introduce an effect of stress concentrators at the top of the graphite slats, which confers lower mechanical strength properties compared to nodular (spheroidal) castings.

Silicon is a graphitizing element and is added to cast iron to promote the separation of carbon in the form of graphite. The higher concentration of silicon in the EN-GJS-400-15 cast iron helped to form the graphite spheres in the cast iron structure. The presence of manganese in cast iron gives it great hardness. Gray cast iron with lamellar graphite type EN-GJL-250 has a higher concentration of manganese (0.354%), so this cast iron is a hard cast iron, but it is more fragile than cast iron with spheroidal graphite EN-GJS-400-15.

## 4. Conclusions

In this paper, it was analyzed the behavior of two types of material, namely the cast iron with lamellar graphite type EN-GJL-250 and cast iron with spheroidal graphite type EN-GJS-400-15, which were cast by gravitational casting method into ceramic molds made by Binder Jetting 3D printing method in the preamble.

A finite element analysis was initially performed using the Abaqus program, in order to study the Tresca and von Mises stresses, as well as the deformations of the material.

Tensile tests were also performed for 20 samples made of EN-GJL-250 and EN-GJS-400-15, cast iron material using ceramic molds manufactured by the 3D printing method to determine their characteristics and to realize morphological analyses at the end. The most important conclusions regarding the activities that were performed regarding this research were the following:

- The results reached after finite element analyses emphasized that when a pressure of 25 MPa is being applied, in the case of the parts made of cast iron with lamellar graphite of type EN-GJL-250 a safety factor of at least 1.5 (normal value used in hydraulic applications) is not guaranteed, this condition being ensured only for cast iron of type EN-GJS-400-15.
- The results of the tensile tests revealed that cast iron with spheroidal graphite type EN-GJS-400-15 has higher tensile strength as compared to the ones made of EN-GJL-250 material, specific deformations that were reached being also much lower in this variant. The samples made of cast iron with spheroidal graphite type EN-GJS-400-15 had an elongation of about 20% before breaking, as compared to those made of cast iron with lamellar graphite type EN-GJL-250 which suffered a sudden fracture.
- SEM analyses performed for the fracture areas of the samples revealed that the EN-GJL-250 sheet metal cast iron is a 100% pearlite cast iron, which makes the resulting parts hard but very fragile, and the spheroidal cast iron of type EN-GJS-400-15 has in its structure approximately 80% ferrite and 20% pearlite, with ferrite giving it higher plasticity. The presence of magnesium concentration in case of EN-GJS-400-15 material is an element that determines the nodular shape of the graphite in the structure, improving its mechanical behavior and characteristics of realized parts at the end.

## Figures and Tables

**Table 1:** Results obtained from the tensile tests of gray cast iron samples with lamellar graphite type EN-GJL-250.

Test No.	Maximum Loading (N)	Specific Deformation (mm)	Tensile Strength (MPa)	Elongation at Break (%)	Elasticity Modulus (MPa)
1	22,103.12	0.01245	275.34	1.9	37,039.12
2	21,457.34	0.01187	273.99	1.56	36,996.34
3	21,333.09	0.01156	273.03	2.04	37,010.34
4	21,074.23	0.01089	273.75	0.99	37,002.67
5	21,692.11	0.01134	271.56	1.03	37,039.11
6	21,902.52	0.01239	273.12	1.79	37,056.19
7	21,221.09	0.01203	271.83	2.98	36,999.13
8	21,739.45	0.01156	273.98	1.24	37,002.19
9	21,492.12	0.01194	273.46	1.2	37,001.85
10	21,503.03	0.01167	274.09	1.45	36,997.45
11	21,543.98	0.01253	275.45	1.67	36,989.73
12	21,703.90	0.01212	274.01	2.05	37,000.00
13	21,442.77	0.01172	273.83	1.08	37,001.14
14	21,389.38	0.01128	274.15	2.06	36,983.18
15	21,406.72	0.01201	273.11	0.95	36,999.87
16	21,632.22	0.01232	275.05	2.43	37,002.04
17	21,541.08	0.01199	273.34	1.56	37,011.01
18	21,320.45	0.01185	272.17	1.77	36,896.98
19	21,711.07	0.01277	273.38	2.81	37,027.01
20	21,210.84	0.01203	273.91	1.11	37,001.06

**Table 2:** Results of tensile tests on EN-GJS-400-15 spheroidal graphite cast iron testing samples.

Test No.	Maximum Loading (N)	Specific Deformation (mm)	Tensile Strength (MPa)	Elongation at Break (%)	Elasticity Modulus (MPa)
1	35,151.12	0.16002	417.60	22.03	54,111.03
2	35,522.63	0.16381	417.57	22.39	54,127.35
3	35,499.02	0.16306	417.57	22.40	54,129.78
4	35,628.14	0.17030	417.58	22.33	54,322.09
5	35,382.70	0.16261	417.56	22.29	54,133.67
6	35,481.90	0.16333	417.58	22.01	54,234.78
7	35,372.71	0.16272	417.58	22.30	54,333.14
8	35,382.12	0.16283	417.58	22.23	54,420.03
9	35,222.01	0.16234	417.59	22.40	54,122.39
10	35,378.92	0.16301	417.59	22.51	54,127.60
11	35,310.37	0.16221	417.58	22.13	54,085.69
12	35,591.25	0.16451	417.59	22.18	54,123.21
13	35,721.03	0.16533	417.57	22.03	54,288.51
14	35,121.17	0.16244	417.57	22.41	54,244.06
15	35,372.31	0.16202	417.58	22.19	54,179.05
16	35,235,.81	0.16281	417.58	22.20	54,190.56
17	35,322.63	0.16325	417.58	22.28	54,088.45
18	35,434.13	0.16298	417.58	22.37	54,031.56
19	35,395.02	0.16182	417.58	22.09	54,100.76
20	35,297.77	0.16204	417.58	22.13	54,108.56

**Table 3:** Material characteristics of the EN-GJL-250 and EN-GJS-400-15 cast iron materials.

Characteristic	Symbol	Measuring Unit	EN-GJL-250	EN-GJS-400-15
Young Module	E	MPa	120,000	120,000
Poisson Ratio	*V*	-	0.26	0.26
Transversal Elasticity Modulus	G	MPa	6500	6500
Density	ρ	$\frac{\mathrm{Kg}}{m^{3}}$	7250	7250
Tensile Strength	$R_{m}$	MPa	250	400
Yield strength	$R_{p0.2}$	MPa	165.59	250
Coefficient of linear thermal expansion	A	$K^{-1}$	1.05 × 10^−5^	1.05 × 10^−5^
Thermal Conductivity	k	$\frac{W}{m\cdot K}$	58	58
Specific heat	cp	$\frac{J}{\mathrm{Kg}\cdot K}$	460	460

**Table 4:** Chemical analysis of the sample made of gray cast iron with lamellar graphite type EN-GJL-250.

**C [%]**	**Si [%]**	**Mn [%]**	**P [%]**	**S [%]**	**Cr [%]**
3.61	2.14	0.156	0.0127	0.0113	0.0320
**Mo [%]**	**Ni [%]**	**Al [%]**	**Cu [%]**	**Nb [%]**	**Ti [%]**
0.00759	0.216	0.0115	0.0707	0.00133	0.00700
**V [%]**	**W [%]**	**Pb [%]**	**Sn [%]**	**Mg [%]**	**+Bi [%]**
0.0105	0.00133	0.00157	0.00442	0.0430	0.00204
**Ca [%]**	**Ce [%]**	**Sb [%]**	**Te [%]**	**B [%]**	**Fe [%]**
0.0060	0.00434	0.0004	0.00176	0.00022	93.6

**Table 5:** Chemical analysis of the sample made of gray cast iron with spheroidal graphite type EN-GJL-400-15.

**C [%]**	**Si [%]**	**Mn [%]**	**P [%]**	**S [%]**	**Cr [%]**
3.34	1.53	0.354	0.0377	0.0990	0.0449
**Mo [%]**	**Ni [%]**	**Al [%]**	**Cu [%]**	**Nb [%]**	**Ti [%]**
0.0115	0.0115	0.0115	0.0115	0.0115	0.0115
**V [%]**	**W [%]**	**Pb [%]**	**Sn [%]**	**Mg [%]**	**+Bi [%]**
0.00895	0.00895	0.00895	0.00895	0.00895	0.00895
**Ca [%]**	**Ce [%]**	**Sb [%]**	**Te [%]**	**B [%]**	**Fe [%]**
0.00036	0.00112	0.0004	0.00192	0.00011	94.2

## Data Availability

Data Sharing is not applicable.
